# Study on Preparation of Long-Afterglow Luminescent Road-Marking Coatings and Simulation of Road Layout

**DOI:** 10.3390/ma19020215

**Published:** 2026-01-06

**Authors:** Xiaowei Feng, Bo Li, Yan Zhang, Yanrong Xu

**Affiliations:** 1 School of Civil Engineering, Lanzhou Jiaotong University, Lanzhou 730070, China; libolzjtu@hotmail.com; 2 Gansu Transportation Science Research Institute Group Co., Ltd., Lanzhou 730070, China; 3 School of Traffic and Transportation, Lanzhou Jiaotong University, Lanzhou 730070, China; zhangyan1978@lzjtu.edu.cn (Y.Z.); 15209627068@163.com (Y.X.)

**Keywords:** luminescent material, road-marking coatings, long afterglow, luminescent performance, Twinmotion simulation, road safety

## Abstract

To improve night-time visibility of pavement markings, a long-afterglow road-marking coating was developed using strontium aluminate as the phosphorescent component. The influences of particle size (100–400 mesh), dosage (15–35 wt%), filler type, and coating thickness (200–600 μm) on optical behavior were systematically evaluated. The optimal formulation—200-mesh strontium aluminate at 30 wt%, titanium dioxides combined with ultrafine glass powder, and a thickness of 500 μm—achieved an initial brightness of 3.08 cd/m^2^ and maintained visible afterglow for more than 9 h. Durability tests confirmed satisfactory resistance to water, alkali, and abrasion, meeting the requirements of JTT 280-2022. Twinmotion simulations further demonstrated that when the coating brightness remains above 0.1 cd/m^2^, it provides effective visual guidance on unlit road sections, thereby enhancing night-time driving safety. This study verifies the feasibility of using long-afterglow coatings to improve road visibility and reduce night-time accident risks.

## 1. Introduction

Road traffic markings serve as visual guides and safety facilities, playing a crucial role in preventing and reducing traffic accidents [1,2]. With increasing public awareness of safety, there is a growing demand for highly visible traffic markings. However, existing markings often exhibit poor visibility at night, which compromises driving safety. Therefore, enhancing the night-time visibility of traffic markings is of great significance.

Currently, common methods to improve marking visibility include the use of glass microspheres on paint surfaces, LED markings, and luminescent coatings. Glass microspheres can enhance visibility under lighting, but their effect is limited in the absence of light [3,4]. LED markings, typically used in city intersections and tidal lanes, are effective but costly for large-scale highway applications [5]. Luminescent markings, on the other hand, utilize materials that absorb natural or artificial light and gradually release it as visible light [6,7]. Luminescent coatings offer high initial brightness and long afterglow durations, guiding drivers’ lines of sight at night, are generally considered to have low toxicity and minimal environmental impact [8].

Luminescent marking coatings generally consist of luminescent materials, film-forming agents, fillers, and additives [9]. Many researchers have investigated the performance of luminescent coatings. For example, Wang et al. [10] prepared long-afterglow coatings using petroleum resin and silicate luminescent materials and studied the effect of luminescent content on coating performance. Cao et al. [11] examined the stability of luminescent markings by varying the pigment-to-binder ratio. Bacero et al. [12] analyzed how illumination time, emission brightness, and doping amount influence coating performance. Bi et al. [13] explored the effects of different acrylic copolymer emulsions and additives on film properties, ensuring compliance with the requirements of basic marking coatings. Xu et al. [14] developed a long-afterglow coating capable of emitting light for over six hours after daytime sunlight exposure. However, many existing luminescent coatings still suffer from low brightness, short afterglow duration, and limited durability, and most studies remain limited to laboratory tests, without evaluating their actual night-time visibility. Therefore, further research is needed to develop higher performance coatings and to assess their practical visual effectiveness.

Common visualization software includes DIA Lux, UC-win/Road, and Twinmotion. DIA Lux, primarily for lighting design, supports the importation of light-source data (e.g., IES, CIB, ULD, and LDT) from major manufacturers [15]. Using DIA Lux, the Sun simulated park road lighting under different conditions, showing that road type, illuminance, and light color significantly affect night-time perceptual preference [16]. UC-win/Road simulates driving conditions and allows for adjustment of parameter settings such as vehicle speed, travel level, and longitudinal clamp angle [17]. Chen used UC-win/Road to evaluate roadside greening schemes at tunnel entrances, confirming effective guidance of drivers’ sightlines [18]. Twinmotion, a 3D visualization tool, was employed in this study to simulate the deployment and luminescence effect of long-afterglow coatings [19,20].

In summary, this work prepared a long-afterglow luminescent coating with high brightness, long service life, and good durability by blending strontium aluminate luminescent material with silicone–acrylate emulsion, titanium dioxide, ultrafine glass powder, and additives. The effects of various factors on afterglow performance were investigated, and coating performance was verified. Finally, Twinmotion was used to simulate the deployment and visual effect of the coating, providing a reference for practical applications of long-afterglow luminescent markings.

## 2. Materials and Test Methods

### 2.1. Experimental Design

**Figure 1:**
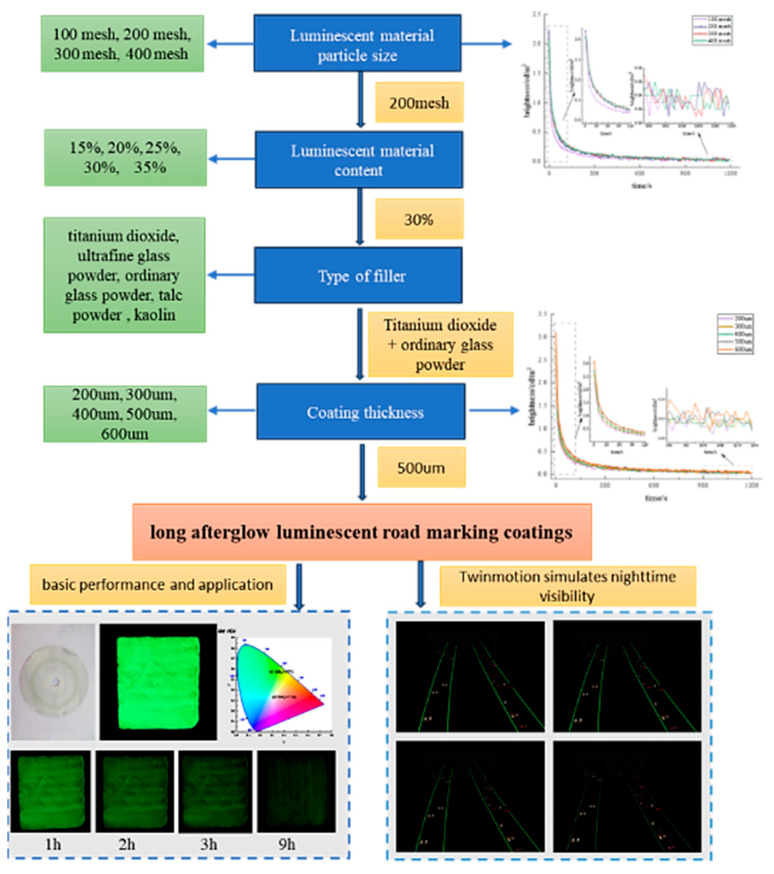
Flow chart of experimental design.

Figure 1 shows the flow chart of the experimental design.

### 2.2. Raw Materials

Long-afterglow luminescent coatings are mainly composed of luminescent materials, film-forming substances, fillers, and additives. The selection of raw materials is described as follows:

(1) Luminescent materials

Luminescent materials are substances that can absorb energy (such as sunlight or artificial light) and release it as visible light, producing a glow. Currently, the most common luminescent materials include sulfide, silicate, aluminate, silica–aluminate, etc. Among them, aluminate luminescent material has high luminescence brightness, a long afterglow time, and good stability [21,22]. Strontium aluminate is the most widely used long-afterglow luminescent material. Strontium aluminate is known to exhibit hydration behavior, which may influence its luminescent and structural properties [23]. Appropriate handling and storage are necessary to maintain its optical performance. Therefore, in this paper, strontium aluminate luminescent materials are studied as luminescent materials for long-afterglow luminescent coatings.

(2) Film-forming substances

Silicon–acrylate emulsion combines the high-temperature resistance, weatherability, and chemical resistance of silicone and the high color retention, flexibility, and adhesion of acrylic resin. Silicon–acrylate emulsion is an environmentally friendly emulsion with high weatherability and water resistance [24]. Therefore, silicon–acrylate emulsion was selected as the film-forming substance for the long-afterglow luminescent coating.

(3) Fillers and additives

Five fillers—talc powder, titanium dioxide, glass powder, kaolin, and ultrafine glass powder—were selected to optimize the coating performance. Talc powder improves surface smoothness, while kaolin enhances brightness and mechanical strength. Titanium dioxide increases light scattering and luminescence visibility, and glass powders improve hardness, wear resistance, and the uniformity of the afterglow. The optimal filler combination was determined based on initial brightness and overall performance. Additives included film-forming agents, defoaming and leveling agents, and fumed silica as an anti-settling agent to maintain uniform dispersion.

Details with respected to suppliers, purities, and batch information of all raw materials used in this study are summarized in Table 1 to ensure the reproducibility of the experiments.

### 2.3. Long-Afterglow Luminescent Coating Preparation

The experiments were prepared according to the basic formulation in Table 2. A proportion of 50–70% of silicone propylene emulsion placed into a beaker and mixed using a magnetic stirrer at 500 rpm for 15 min to ensure uniform dispersion. A measured amount of fumed silica was then added and stirred under the same conditions until evenly distributed. Luminescent materials and fillers were slowly incorporated into the mixture and continuously stirred at 500 rpm for 15 min. Finally, additives were added, and the mixture was stirred at the same speed and duration until a homogeneous, light-white long-afterglow luminescent coating was obtained. The selected raw materials were mainly sourced from Badefu Technology Co., Ltd. (Guangdong, China). and Anhui Annada Taiye Co., Ltd. (Anhui, China).

### 2.4. Characterization of Long-Afterglow Luminescent Coatings

Luminescence brightness test: The long-afterglow luminescent coating was coated on uralite with a paint-film coater. Then, the coated test plate was put into a dark room for 24 h before the test. After 20 min of excitation under a standard D65 light source, the light source was removed. The luminance of the coating film was measured using a CS-200 luminance meter (Tokyo, Japan).

Afterglow time test: The luminescent coating was painted onto the cement specimen evenly, absorbing the external light source, from excitation to attenuation to the minimum luminance visible to the human eye (0.32 mcd/m^2^) until it was not perceived by the human eye, and the duration of the afterglow was recorded.

To quantitatively describe the afterglow attenuation behavior of the long-afterglow luminescent coatings, the luminescence decay curves were fitted using the commonly applied multi-exponential decay model for SrAl_2_O_4_^2+^, Dy^3+^-based phosphors. Strontium aluminate materials typically exhibit multiple trapping depths and thermally stimulated detrapping processes; therefore, their decay behavior cannot be accurately represented by a single-exponential model. A double-exponential luminescence decay equation, as widely adopted in previous studies on strontium aluminate phosphors, was used to characterize the temporal evolution of brightness, as shown in Equation (1) [25]:

(1) $$I(t)=I_{1}e^{-t/\tau_{1}}+I_{2}e^{-t/\tau_{2}}$$

where *I*(*t*) represents the luminescence intensity at time *t*; *I*_1_ and *I*_2_ are the initial intensities of the fast and slow decay components, respectively; and *τ*_1_ and *τ*_2_ are the corresponding decay time constants. The fast component (*τ*_1_) is associated with shallow traps, while the slow component (*τ*_2_) represents the deeper traps that dominate long-duration afterglow. This model enables accurate characterization of the afterglow properties and allows for comparison of material performance across different coating formulations.

Coating performance test: The properties of long-afterglow luminescent coatings were tested according to the JTT280-2022 pavement-marking paint specification (in China) [26], including the coating in the container in terms of condition, appearance, water resistance, and abrasion resistance.

### 2.5. Twinmotion Simulation

Twinmotion 2023.x is the most widely used 3D visualization and real-time rendering 3D software. In practice, the unreal engine is used as the core engine to efficiently complete video and image processing and production to ensure the quality of the final production [27].

In order to investigate the night-time visibility of long-afterglow luminescent coatings, 3D Studio Max (3ds Max 2023) was used to construct a highway model. The model was imported into Twinmotion; the materials were replaced; the appropriate natural and artificial lighting conditions, etc., were selected; and the video footage, pictures, or moving images were exported. Laying luminescent coatings of different brightnesses to simulate the visual recognition effect at night guides the line of sight and, thus, reduces the rate of traffic accidents at night. Brightness was adjusted mainly by assigning a material to the texture using the luminescence parameter in the material properties. The luminance parameters used for subsequent simulations were derived from experimental data.

## 3. Results and Discussion

### 3.1. Influence of Different Factors on the Luminescence Performance of Coatings

#### 3.1.1. Luminescent Material Particle Size

**Figure 2:**
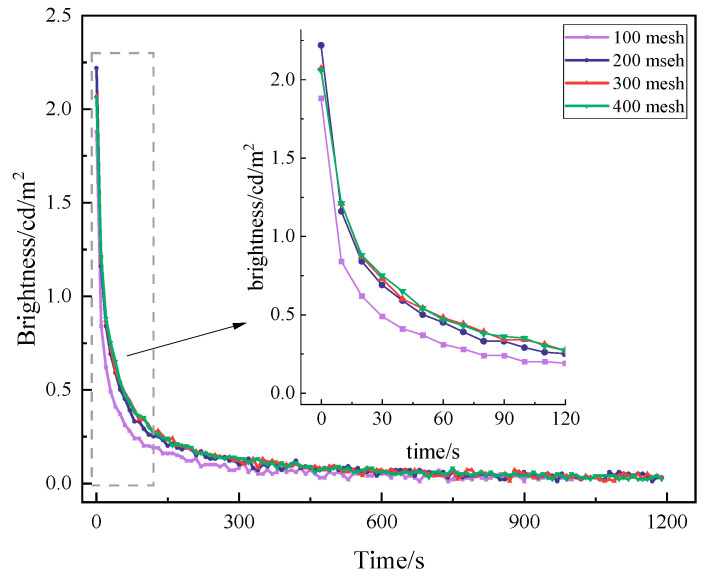
Afterglow decay curves of luminescent coatings with different particle sizes.

The particle size has a great influence on the luminescence performance and coating performance of long-afterglow luminescent materials. Larger phosphor particles produced higher initial brightness and longer afterglow duration after excitation but also resulted in rougher film surfaces and increased sedimentation. However, if the particle size of the luminescent material is too large, a certain degree of precipitation will occur during the storage of the coating, as well as during the coating process. Meanwhile, the rough surface and uneven distribution after film formation affect the luminescence performance of the coatings to different degrees [28]. Therefore, a particle size gradient test was carried out in this study, and the same luminescent material with four different particle sizes (100 mesh, 200 mesh, 300 mesh, and 400 mesh) was used. According to the same mass ratio, three coating samples were prepared for each group of coatings, then irradiated by a D65 standard light source for 20 min, and their luminous properties were tested by a CS-200 chrominance meter. The results are shown in Figure 2.

As can be seen in Figure 2, the decay curves of the luminescent coatings containing luminescent materials of different particle sizes have the same trend of both fast and slow decay stages. In the initial 10 min of decay is faster, from the initial brightness decay to 0.1 cd/m^2^ or less, and 10 min after the decay rate is slow, then slowly leveling off. When the particle size of the luminescent material is 100 mesh, the initial brightness is only 1.88 cd/m^2^. The luminescence brightness can only be collected to the brightness of 16 min after the withdrawal of the light source (0.02 cd/m^2^), and the rest of the grain size can be collected up to 20 min or even longer. The coating brightness of the luminescent material with a particle size of 200 mesh had a relatively high brightness, with an initial brightness of 2.22 cd/m^2^, and the brightness decreased to 0.03 cd/m^2^ after 20 min. It can be seen that the particle size has a certain effect on the luminescence brightness. In other words, large-particle-size luminescent materials have a higher brightness. However, the surface of a luminescent coating containing too large of a particle size is rough, unevenly distributed, and easy to precipitate, which are properties not conducive to light output. On the other hand, when the particle size is small, the initial brightness of the luminescent coating will be affected to a certain extent. Therefore, for luminescent coatings, the particle size should be appropriate. Considering the initial brightness, film-forming property, transmittance, and other factors, it was determined to use 200-mesh luminescent material as the long-afterglow light source.

#### 3.1.2. Luminescent Material Content

**Figure 3:**
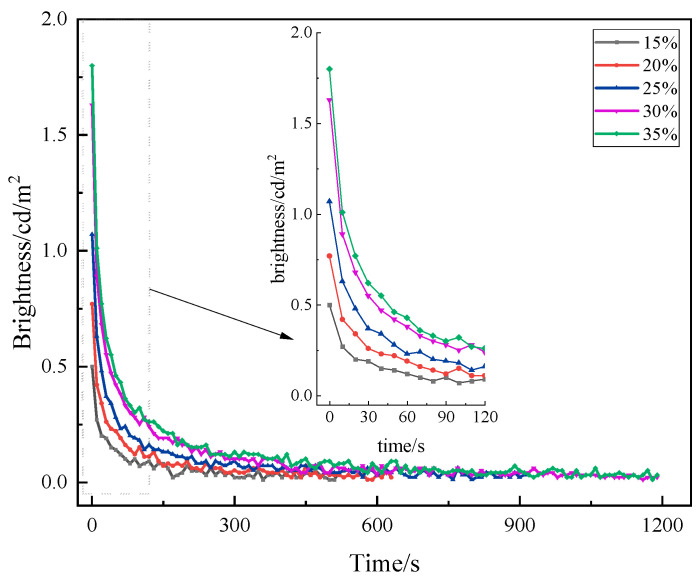
Afterglow decay curves of luminescent coatings with different amounts of luminescent materials.

The content of luminescent materials in long-afterglow luminescent coatings has a large impact on the luminescence brightness [29]. The higher the content, the more luminescent material per unit area and the better the luminescence effect. However, excessive phosphor loading leads to particle stacking, which reduces luminous efficiency and weakens the brightness. Therefore, a content gradient test of luminescent materials was carried out to prepare luminescent coatings using five contents (15%, 20%, 25%, 30%, and 35%) of luminescent materials. The luminescence performance was tested, and the results are shown in Figure 3.

As can be seen in Figure 3, the five curves in the graph follow roughly the same trend. With the increase in amount of luminescent materials, the initial brightness gradually increases, and the growth rate is high. This is because as the number of luminescent centers in the luminescent coating is increased, the spacing of each luminescent center becomes smaller, and the interaction is enhanced. When the content of luminescent material is lower than 30% (mass fraction), the initial brightness is below 1.5 cd/m^2^. When the content of luminescent material is 30%, the luminescence performance of the prepared luminescent coating is better, with an initial brightness of 1.63 cd/m^2^. When 35% luminescent material is added, the luminescent performance of the coating increases slightly—only by 0.17 cd/m^2^. This is because the number of luminescent centers is higher and the energy is saturated if the content of luminescent materials is higher. For luminescence attenuation, when the content of luminescent material is lower than 30%, the brightness of the luminescent coating after the stopping of irradiation for 20 min cannot be measured. When the content was increased to 30% and 35%, the brightness decayed to 0.02 cd/m^2^ and 0.03 cd/m^2^ after 20 min, respectively. In addition, when the content of luminescent material is high due to the high density of luminescent material, it is easy to precipitate, which affects the storage stability of luminescent coatings. Moreover, the use of too much luminescent material will increase the cost. Therefore, it is recommended that the content of luminescent materials be 30%.

#### 3.1.3. Type of Filler

**Figure 4:**
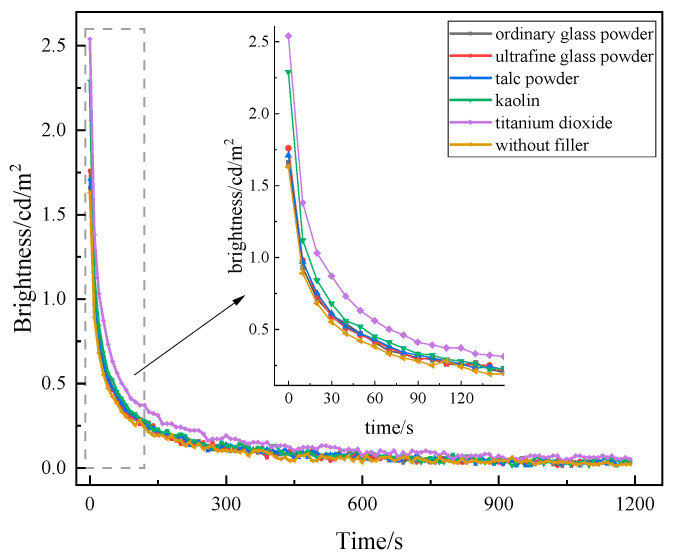
Effect of filler on the afterglow decay curve of luminescent coating.

At present, the most common fillers on the market in China are generally white or slightly colored powders, and different fillers have different effects on the luminescence performance of long-afterglow luminescent coatings. Five kinds of commonly used fillers were selected—titanium dioxide, ultrafine glass powder, ordinary glass powder, talc powder, and kaolin—at a content of 10%. The preparation of long-afterglow luminescent coating was tested for luminescence performance. The test results are shown in Figure 4.

**Figure 5:**
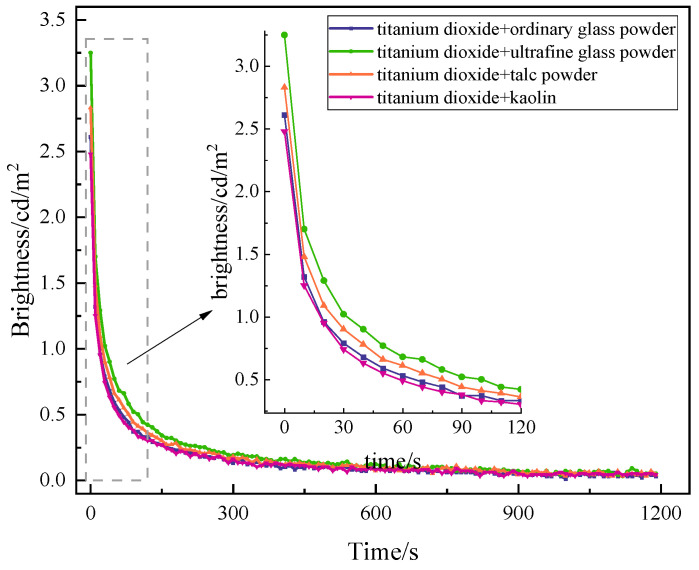
The afterglow decay curve of luminescent coating with mixed fillers.

As can be seen in Figure 4, the trend of the afterglow decay curves of the five fillers is consistent, and the initial brightness is higher than that of 1.63 cd/m^2^ when no filler is added. Among them, the initial brightness of titanium dioxide increased the most, with an increase of 56%. The addition of kaolin improves the initial brightness of the luminescent coatings as well, but the effect is not as pronounced as that of titanium dioxide. The rate of increase of ordinary glass powder, ultrafine glass powder, and talc powder is relatively small. Rutile titanium dioxide enhances the brightness of the coating due to its high refractive index, while its dense structure provides strong UV resistance and stable chemical properties [30]. However, due to the slightly higher price of titanium dioxide, in order to saving costs, titanium dioxide and filler, mixed at a mass ratio of 1:1, were added to the long-afterglow luminescent coatings and tested for luminescence performance. The test results are shown in Figure 5.

As can be seen in Figure 5, compared with coatings containing a single titanium dioxide, the initial brightness of luminescent paint containing mixed filler exhibits a certain degree of improvement. When luminescent paint contains titanium dioxide mixed with kaolin filler, there is a slight decrease in the initial luminous brightness but no significant decline. The luminous paint containing a combination of titanium dioxide and ultrafine glass powder exhibits the largest initial brightness increase of 28%, and luminous paint containing titanium dioxide combined with talcum powder filler results in an increase in initial brightness of 11%. When the excitation light was stopped for 20 min, the brightness of the coatings with the combination filler of titanium dioxide and ultrafine glass powder combination decayed to 0.05 cd/m^2^. The brightness of coatings with a combination of titanium dioxide and ordinary glass powder fillers decayed to 0.03 cd/m^2^ after the light source was withdrawn for 20 min. The brightness of the coatings with titanium dioxide and talc powder combination filler and titanium dioxide and kaolin combination filler both decayed to 0.04 cd/m^2^ after the light source was withdrawn for 20 min. The combination of titanium dioxide and ultrafine glass powder results in a greater increase in initial brightness, and the 20 min brightness is also higher than that of other types of combination filler. This is because the paint film formed after the dispersion of ultrafine glass powder, the coating is transparent, and the opacity is close to that of emulsion. Light passes through these two materials without diffuse reflection, showing better light transmission and reflectivity [26]. At the same time, due to the small amount of the addition and its better light transmittance, when the coating is thin, ordinary glass powder alone cannot play its effect well. Therefore, when it is combined with titanium dioxide, the initial brightness is significantly increased.

#### 3.1.4. Coating Thickness

**Figure 6:**
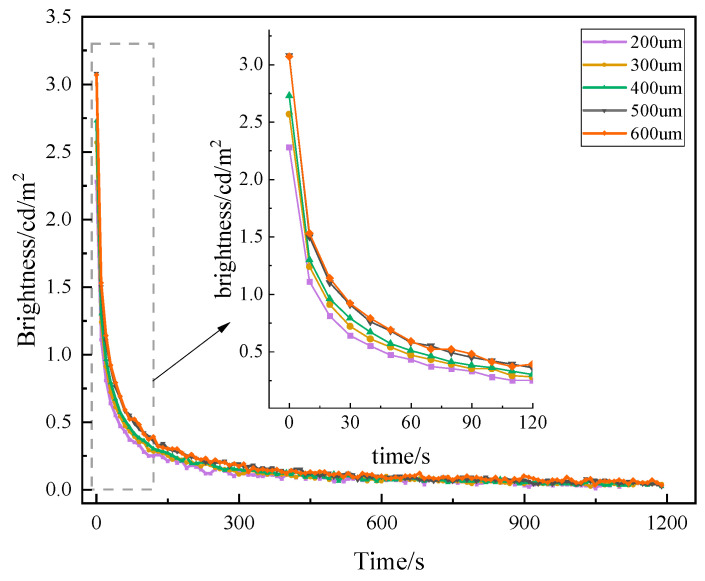
Afterglow decay curves of luminescent coatings with different coating thicknesses.

After raw materials, such as luminescent materials, as well as fillers, are determined, the effect of the coating thickness on the luminescent performance also needs to be considered [31]. As the thickness of the coating increases, the luminescent material per unit area will be aggregated, the luminescent center is increased, and the luminescence brightness will be higher. When the thickness reaches a certain level, the luminescent material will produce a buildup between the stacking and superposition so that the contribution of the luminescent material at the bottom of the coating is reduced, which, in turn, affects the glowing effect. This paper compares the luminescence performance of the same luminescent coating with five thicknesses (200 μm, 300 μm, 400 μm, 500 μm, and 600 μm), and the test results are shown in Figure 6.

As can be seen in Figure 6, the decay trend of luminescence brightness of luminescent coatings with different coating thicknesses over time is basically the same over 20 min. The initial brightness increases with the increase in coating thickness, and when the thickness increases to a certain value, the luminescence brightness increases slowly. This is because when the coating thickness is moderate, the luminescent material in the coating can reach the optimal utilization rate, fully absorbing the external light source, resulting in a good luminescence effect. However, as the coating thickness increases, the obstruction of light by film-forming substances, luminescent materials, and fillers increases. The luminescent material at the bottom of the coating cannot fully absorb the external light source, resulting in a reduced contribution of the luminescent material at the bottom to the initial brightness of the coating. As a result, the initial brightness no longer increases significantly beyond this thickness because internal layers obstruct light absorption. When the thickness of more than 500 um, the amplitude of the increase in the initial brightness of the coating is lower, so the utilization of luminescent material is higher when the coating thickness is 500 um.

### 3.2. Basic Performance and Application of Long-Afterglow Luminescent Marking Coating

(1) The basic performance of long-afterglow luminescent marking coating

**Figure 7:**
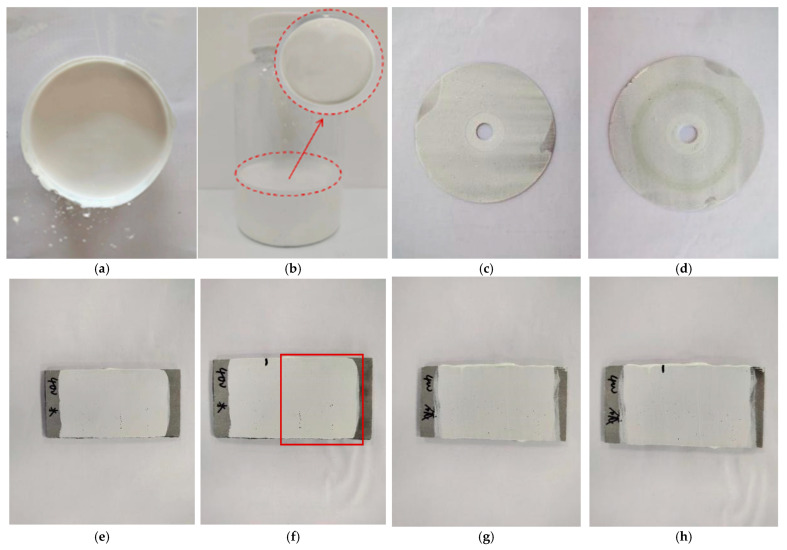
Basic performance test diagram of long-afterglow luminescent marking coating. (**a**) Initial state of coating. (**b**) Status after 15 days. (**c**) Before abrasion. (**d**) After abrasion. (**e**) Before water resistance. (**f**) After water resistance. (**g**) Before alkali resistance. (**h**) After alkali resistance.

The long-afterglow coating exhibited good storage stability, showing no aggregation in the container and remaining easy to stir. Following static deposit of the Luminescent coating for 15 days, no aggregation or stratification was observed, that is, the stability of the luminescent marking coating was better, as shown in Figure 7a,b. Figure 7c,d show the samples of abrasion. The abrasion value of the marking coating is 3 mg, which is almost no abrasion and meets the specification requirements. Figure 7e is the coating before water resistance. The right side of Figure 7f is the state after soaking in aqueous solution for 48 h. It can be seen that the preparation coating has no abnormality after soaking in water. Figure 7g shows the coating before alkali resistance. The right side of Figure 7h shows the state of the coating after being immersed in calcium hydroxide-saturated solution for 24 h. It can be seen there is no abnormality in the coating after immersion. The water resistance and alkali resistance are good, meeting the requirements of practical application.

(2) Chromaticity of long-afterglow luminescent marking coating

**Figure 8:**
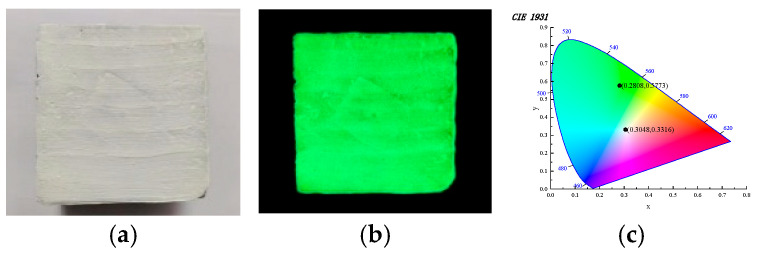
Chromaticity diagram of long-afterglow luminescent marking coating. (**a**) Specimen before absorbing light. (**b**) After the specimen absorbs light. (**c**) Color coordinate diagram.

The coatings were applied to the cement specimens and tested for their chromatic properties after 24 h of curing, as shown in Figure 8. In Figure 8a,b, it can be seen that the coating formed a white film on the surface of the cement after curing. When the specimen is excited by the light source, a green light is emitted, which is consistent with the test results in Figure 8c. Green light is the most sensitive light to the human eye among the three primary colors, indicating that the prepared coatings can be well captured by the human eye. Therefore, the prepared long-afterglow luminescent coating can be used for road traffic marking and other scenes to ensure the safety of night travel.

(3) Aging properties of long-afterglow luminescent marking coating

**Figure 9:**
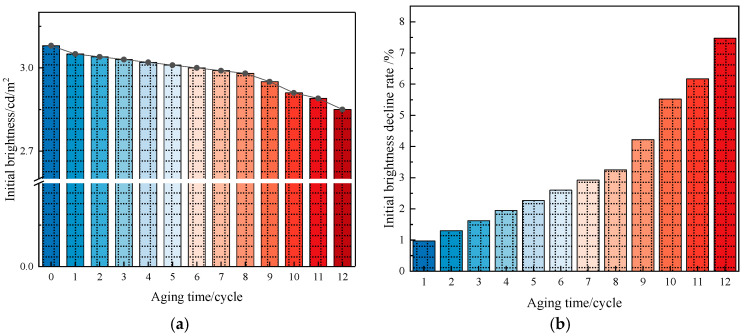
Brightness change before and after aging. (**a**) Initial brightness statistics. (**b**) Initial brightness decline-rate statistics.

**Figure 10:**
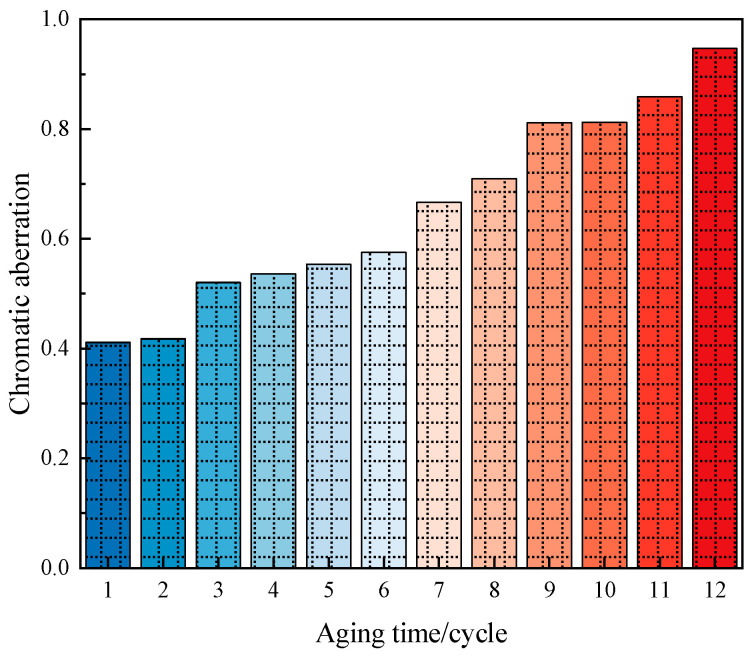
Chromatic aberration before and after aging.

Long-afterglow luminescent marking coating is exposed to the natural environment, aging under the action of sunlight, oxygen, and water. However, the aging cycle of coatings studied under natural conditions is relatively long; therefore, a laboratory was used to simulate the aging of coatings under natural conditions. A UVA-340 ultraviolet lamp (Wuhan Shangce Testing Equipment Co., Ltd., Wuhan, China) was selected as the light source-generating device to simulate summer noon sunlight exposure, as it can adequately simulate the critical short-wave wavelength range of the solar spectrum. The wavelength range was 320~400 nm, and light-emitting spectral energy was mainly concentrated in the 340 nm wavelength. The experimental parameters for accelerated aging were as follows: a cycle consisted of two repetitions of an 8 h UV irradiation phase (0.51 W/m^2^, UVA-340 lamp) at 60 °C and a 4 h dark condensation phase at 50 °C. Thus, each full cycle had a duration of 24 h. A total of 12 such cycles were conducted, equivalent to 288 h of continuous testing. The initial brightness decline rate and chromatic aberration were calculated using Formulas (2) and (3). The calculation results are shown in Figure 9 and Figure 10.

(2) $$X=\frac{A-B}{A}*100\%$$

where *X* is the initial brightness decline rate of the long-afterglow luminescent coating, *A* is the initial brightness of the coating before the aging test, and *B* is initial brightness of the coating after the aging test.

(3) $$\Delta E=\sqrt{{\left(L_{1}-L_{0}\right)}^{2}+{\left(a_{1}-a_{0}\right)}^{2}+{\left(b_{1}-b_{0}\right)}^{2}}$$

where ΔE is chromatic aberration; *L* is the brightness value; *a* represents red and green values; *b* corresponds to yellow and blue values; *L*_0_, *a*_0_, and *b*_0_ are color parameters of coatings before the aging test; and *L*_1_, *a*_1_, and *b*_1_ are color parameters of the coating after the aging test.

The changes in initial brightness for different aging times can be seen in Figure 9. In Figure 9a, it can be seen that the brightness shows a decreasing trend as the aging time increases, but the attenuation is relatively small. It can be seen in Figure 9b that as the aging time increases, the initial brightness decline rate shows an upward trend. The change in the initial brightness decline rate at 8 cycles of aging was small, at only 3.25%. The initial brightness decline rate increased in the last four cycles and was 7.47% after 12 cycles of aging, corresponding to less than a 10% reduction in the initial brightness of the coating after aging. This shows that ultraviolet aging has a minimal effect on the luminescent performance of luminescent coatings and that they can be used normally.

As can be seen in Figure 10, the chromatic aberration shows an increasing trend as the aging time increases. After 12 cycles of aging, the Δ*E* values of the coatings are all below 1, which is well below the standard perceptible threshold (Δ*E* > 2.3 [32]) for human vision. Therefore, the color change is minimal and may only be slightly noticeable to individuals with high visual sensitivity, while it is imperceptible to those with normal sensitivity. This indicates that the coatings have good weathering resistance.

(4) Persistent luminescence duration of long-afterglow luminescent marking coating

**Figure 11:**
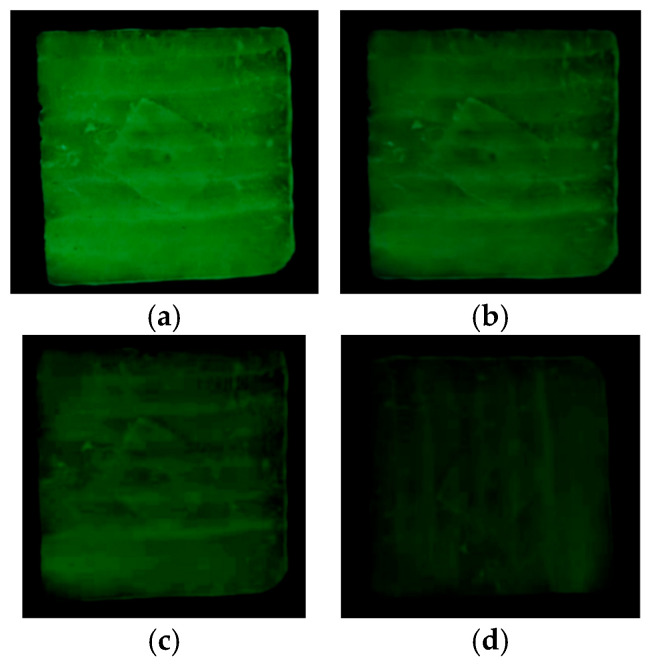
The duration of continuous luminescence of the specimen in a dark environment. (**a**) 1 h. (**b**) 2 h. (**c**) 3 h. (**d**) 9 h.

At the same time, the coating was applied on the cement specimen to observe the duration of continuous luminescence in a dark environment at different times. The results are shown in Figure 11.

As can be seen in Figure 11, the specimen can continue to glow after being irradiated by a standard light source for 20 min. The brightness continues to decay with time. The brightness was higher in the first two hours, and only a weak light was observed in the dark environment at 9 h. The minimum brightness visible to the human eye is 0.32 mcd/m^2^, and the afterglow time of the long-afterglow markers can be seen up to 9 h.

### 3.3. Layout Effect Simulation of the Night Visibility of Long-Afterglow Luminescent Coatings 

**Figure 12:**
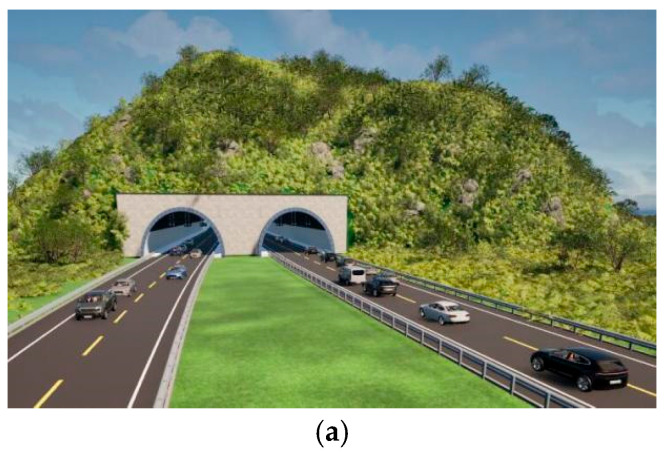

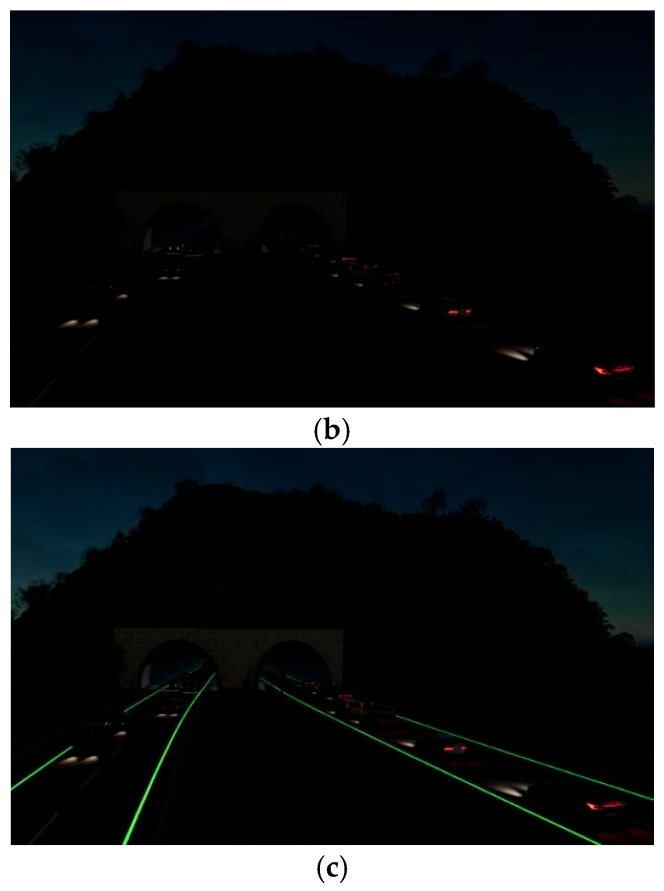
Guidance effects of road markings at different times. (**a**) Daytime—13:00 p.m. (**b**) Night-time—22:00 p.m. (**c**) 22:00 p.m.—state of laying of luminous markings.

In order to investigate the night-time visibility of long-afterglow luminescent coatings, a visual simulation of luminous road-marking coatings was carried out using Twinmotion. Figure 12a shows a scenario at 13:00 p.m. during the daytime, and Figure 12b shows ta scenario at 22:00 p.m. on a highway without illumination. Figure 12c shows the deployment of the long-afterglow luminescent road-marking coatings at 22:00 p.m.

**Figure 13:**
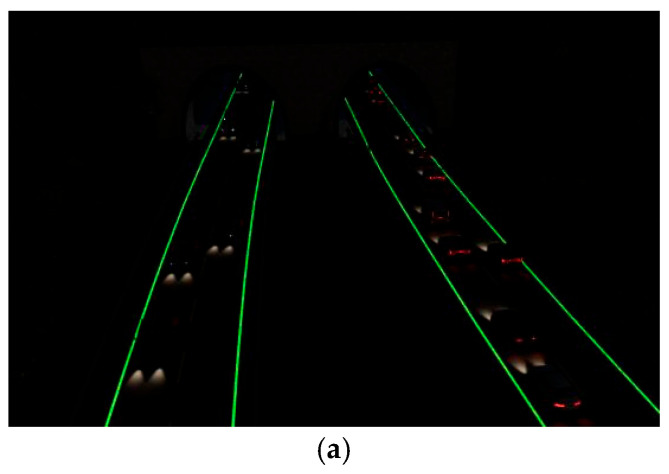

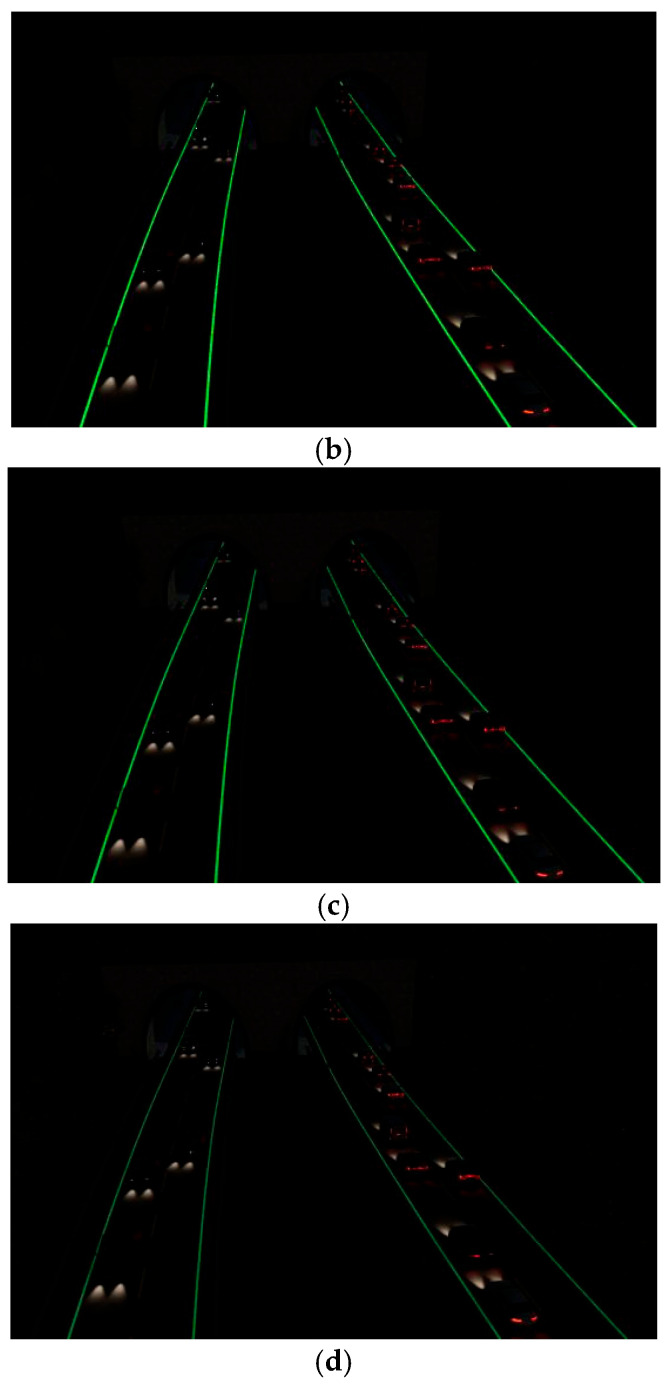
Night road simulation under different marking brightnesses. (**a**) Road conditions with brightness of 3.08 cd/m^2^. (**b**) Road conditions with brightness of 2 cd/m^2^. (**c**) Road conditions with brightness of 1 cd/m^2^. (**d**) Road conditions with brightness of 0.1 cd/m^2^.

It can be seen in Figure 12a that the road traffic markings guide the line of sight during the daytime, and the visibility is high, which can guide the line of sight and regulate driving. Figure 12b shows a scene at night when there are no street lamps or other lighting facilities; it can be seen that the visual recognition of road traffic markings is obviously reduced, the induction effect is poor, and it is difficult to ensure traffic safety. Therefore, it is necessary to install long-afterglow luminescent road-marking coatings on road sections without lighting facilities or dangerous road sections. Figure 12c shows the deployment of long-afterglow luminescent road-marking coatings at night. It can be seen that it is easier to distinguish the direction and shape of the road after deployment, which improves drivers’ attention to road conditions. In order to compare the luminance attenuation of luminescent coatings at night, the effect of luminance coatings at night under different luminance states was simulated. The simulation effect is shown in Figure 13. The choice of simulated brightness is based on the test results of the luminescent properties of the prepared luminescent coatings. The brightness parameters are as follows: the optimal initial brightness is 3.08 cd/m^2^, intermediate brightnesses are 2 cd/m^2^ and 1 cd/m^2^, and 10 min brightness is 0.1 cd/m^2^.

Figure 13a–d show the effect of laying the luminescent road-marking coatings under different brightnesses at 22:00 p.m. Figure 13a simulates the optimal brightness (3.08 cd/m^2^) of the coatings and shows that night-time visibility is good. The brightness simulated in Figure 13b,c is also good for improving visual recognition and safe travel at night. The simulated brightness in Figure 13d is 0.1 cd/m^2^. Compared with other brightnesses, the guiding effect is general. Although this luminance is far higher than the human visual threshold (0.32 mcd/m^2^), it remains sufficient to provide visual guidance on roads lacking lighting facilities. Although the brightness of the luminescent coatings decays faster in the early stage, it decays slowly in the later stage and tends to be stable when it decays to 0.01 cd/m^2^. Vehicle headlights can also excite the coating during night-time driving, allowing the luminescent material to absorb and store light energy for continued emission, ensuring that the luminous brightness is stabilized at more than 0.1 cd/m^2^, that is, the self-made long-afterglow luminescent marking coatings can guide the line of sight and ensure the safety of driving at night.

It should be noted that the luminescent performance and visibility of the coatings in practical road applications may be influenced by ambient lighting, vehicle headlights, and weather conditions such as rain or fog. While the present study evaluates performance under controlled laboratory conditions, field testing under varying environmental conditions would provide a more comprehensive understanding of real-world effectiveness.

## 4. Conclusions

In this study, a long-afterglow luminescent coating was proposed by using strontium aluminate as the functional material and testing the optical properties of the coating by changing different influencing factors. The basic properties and applications of the long-afterglow luminescent marking coating were also analyzed. Finally, a simulation of night-time visibility of long afterglow-luminescent coatings was carried out in conjunction with Twinmotion. The following main conclusions were obtained:

(1) Long-afterglow luminescent coatings were prepared using strontium aluminate as a luminescent material and silicon–acrylic emulsion as film-forming substance, with the addition of fillers and additives. The prepared coating can continue to emit light for more than 9 h after being excited by a light source.
(2) The initial luminance of the long-afterglow luminescent coating can reach up to 3.08 cd/m^2^. The particle size of the luminescent material is 200 mesh, the content is 30%, with titanium dioxide and ultrafine glass powder as the filler, and the thickness of the coating is 500 μm.
(3) Long-afterglow luminescent coatings have better water resistance, abrasion resistance, and weathering resistance than other coatings; meet the use requirements of road markings; and can be used for road signs and other signs.
(4) Twinmotion was used to construct a road scenario without lighting facilities and simulate the deployment of long-afterglow luminescent markings. The simulated optimal brightness of 3.08 cd/m^2^ has a good guiding effect, and attenuation of brightness to 0.1 cd/m^2^ can still guide the line of sight to ensure driving safety.

## Figures and Tables

**Table 1:** Suppliers and specifications of raw materials.

Category	Material	Chemical Name (Full Name)	Purity/Specification	Supplier (China)
Luminescent material	Strontium aluminate phosphor	SrAl_2_O_4_: Eu^2+^, Dy^3+^ long-afterglow phosphor	≥99.9% (trace metals basis)	Shanghai Jinghui Industrial Co., Ltd. (Shanghai, China)
Film-forming substance	Silicone–acrylate emulsion	Silicone–acrylate copolymer emulsion	Solid content 50 ± 1%	Guangdong Bardese Chemical Co., Ltd. (Guangdong, China)
Filler	Talc powder	Hydrated magnesium silicate	≥99%	Liaoning Ketai Mining Co., Ltd. (Liaoning, China)
Filler	Titanium dioxide (rutile)	TiO_2_ (rutile phase)	≥98%, R-902 or equivalent	Anhui Tianyu Titanium Industry Co., Ltd. (Anhui, China)
Filler	Glass powder	SiO_2_-based glass powder	≥99%	Jiangxi Huabo Materials Co., Ltd. (Jiangxi, China)
Filler	Kaolin	Hydrated aluminum silicate	≥98%	Guangdong Kaolin Co., Ltd. (Guangdong, China)
Filler	Ultrafine glass powder	Ultrafine SiO_2_ glass powder	≥99%	Jiangxi Huabo Materials Co., Ltd. (Jiangxi, China)
Additive	Fumed silica	SiO_2_ fumed silica	≥99.8%	Nanjing Youke Nanomaterials Co., Ltd. (Nanjing, China)
Additive	Defoamer	Polyether-modified silicone defoamer	Industrial grade	Guangzhou Demai Chemical Co., Ltd. (Guangzhou, China)
Additive	Leveling agent	Polyacrylate leveling agent	Industrial grade	Guangzhou Demai Chemical Co., Ltd. (Guangzhou, China)

**Table 2:** Basic formulation of long-afterglow luminescent coating.

Material	Recommended Dosage/% (Mass Fraction)
Film-forming substances	50~70
Luminescent materials	15~35
Fillers	10
Film-forming additive	2.4
Anti-settling agents	0.6
Defoaming agent	0.8
Leveling agents	0.8

## Data Availability

The raw data supporting the conclusions of this article will be made available by the authors on request.
